# Identification of CENPM as a key gene driving adrenocortical carcinoma metastasis via physical interaction with immune checkpoint ligand FGL1

**DOI:** 10.1002/ctm2.70182

**Published:** 2025-01-08

**Authors:** Cunru Zou, Yu Zhang, Chengyue Liu, Yaxin Li, Congjie Lin, Hao Chen, Jiangping Hou, Guojun Gao, Zheng Liu, Qiupeng Yan, Wenxia Su

**Affiliations:** ^1^ Department of Physiology School of Basic Medicine Shandong Second Medical University Weifang China; ^2^ Department of Pathology Affiliated Hospital of Shandong Second Medical University Weifang China; ^3^ Department of Ophthalmology Shandong Provincial Hospital Affiliated to Shandong First Medical University Jinan China; ^4^ Department of Urology Surgery Affiliated Hospital of Shandong Second Medical University Weifang China; ^5^ Department of Urology Surgery Shandong Provincial Hospital Affiliated to Shandong First Medical University Jinan China; ^6^ Department of Teaching and Research Section of Introduction to Basic Medicine School of Basic Medicine Shandong Second Medical University Weifang China; ^7^ Neurologic Disorders and Regenerative Repair Lab of Shandong Higher Education Shandong Second Medical University Weifang China

**Keywords:** adrenocortical carcinoma, CENPM, FGL1, metastasis

## Abstract

**Background:**

Distant metastasis occurs in the majority of adrenocortical carcinoma (ACC), leading to an extremely poor prognosis. However, the key genes driving ACC metastasis remain unclear.

**Methods:**

Weighted gene co‐expression network analysis (WGCNA) and functional enrichment analysis were conducted to identify ACC metastasis‐related genes. Data from RNA‐seq and microarray were analyzed to reveal correlations of the *CENPM* gene with cancer, metastasis, and survival in ACC. Immunohistochemistry was used to assess CENPM protein expression. The impact of CENPM on metastasis behaviour was verified in ACC (H295R and SW‐13) cells and xenograft NPG mice. DIA quantitative proteomics analysis, western blot, immunofluorescence, and co‐immunoprecipitation assay were performed to identify the downstream target of CENPM.

**Results:**

Among the 12 035 analyzed genes, 363 genes were related to ACC metastasis and *CENPM* was identified as the hub gene. CENPM was upregulated in ACC samples and associated with metastasis and poor prognosis. Knockdown of *CENPM* inhibited proliferation, invasion, and migration of ACC cells and suppressed liver metastasis in xenograft NPG mice. Collagen‐containing extracellular matrix signalling was primarily downregulated when *CENPM* was knocked down. FGL1, important components of ECM signalling and immune checkpoint ligand of LAG3, were downregulated following *CENPM* silence, overexpressed in human advanced ACC samples, and colocalized with CENPM. Physical interaction between CENPM and FGL1 was identified. Overexpression of *FGL1* rescued migration and invasion of *CENPM* knockdown ACC cells.

**Conclusions:**

*CENPM* is a key gene in driving ACC metastasis. CENPM promotes ACC metastasis through physical interaction with the immune checkpoint ligand FGL1. CENPM can be used as a new prognostic biomarker and therapeutic target for metastatic ACC.

**Highlights:**

- *CENPM* is the key gene that drives ACC metastasis, and a robust biomarker for ACC prognosis.
- Silencing *CENPM* impedes ACC metastasis in vitro and in vivo by physical interaction with immune checkpoint ligand FGL1.
- FGL1 is overexpressed in ACC and promotes ACC metastasis.

## Graphical abstract

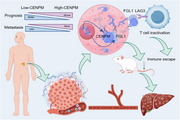

## 1 INTRODUCTION

Adrenocortical carcinoma (ACC) is a rare endocrine malignancy arising in the adrenal cortex, with an incidence of .7–2.0 cases/million people per year.^1^ Distant metastasis occurs in 66% of ACC cases. The lungs, liver, and bone are the most frequently affected organs.^2^ Tumour metastasis is the strongest indicator of poor prognosis.^3^ The median overall survival time for metastatic ACC is less than 1 year^1^, ^2^ and the 5‐year survival is 0–28%.^4^, ^5^, ^6^ However, the key genes driving ACC metastasis remain unclear.

Chromosomal alterations are important biological markers for diagnosis, prognosis, disease classification, risk stratification, and treatment selection in ACC patients.^6^ Chromosomal gains, losses, and loss of heterozygosity are frequently observed in ACC genomes. Somatic inactivation mutation of *TP53*, activation mutation of *CTNNB1*, deletion mutation of *ZNFR3*, and amplification of *TERT* are the most frequent driver mutations in primary ACC.^7^, ^8^, ^9^ However, metastatic ACC has a higher genome‐wide alteration rate and tumour heterogeneity than primary ACC.^1^

In eukaryotes, accurate chromosome segregation during mitosis and meiosis is essential for maintaining genomic stability.^10^ During cell division, an elaborate multiprotein superstructure, the kinetochore, assembles on the centromere and binds spindle microtubules to segregate the replicated chromosomes into daughter cells.^11^ The core proteins required for kinetochore formation include centromere protein A (CENPA), CENPC, and constitutive centromere associated network (CCAN) complexes: CENPL/N, CENPH/I/K/M, CENPO/P/Q/R/U, and CENPT/W/S/X.^11^, ^12^, ^13^ Dysregulation of CENPs promotes aneuploidy with karyotypic heterogeneity, resulting in chromosomal instability (CIN) with lagging chromosomes and micronuclei.^14^, ^15^, ^16^ CENPs are dysregulated in various cancers, showing a significant correlation with disease progression and prognosis.^17^, ^18^, ^19^ In this study, we identified *CENPM* as the key gene in driving ACC metastasis, CENPM promoted metastasis through physical interaction with immune checkpoint ligand FGL1.

## 2 MATERIAL AND METHODS

### 2.1 Datasets

The RNA sequencing (RNA‐seq) data of 77 ACC samples (TCGA) and 128 normal adrenal glands (GTEx) were both downloaded from the UCSC Xena database (https://xena.ucsc.edu/). Four ACC mRNA expression series (project ID: GSE10927, GSE75415, GSE12368, and GSE143383) were acquired from the GEO (http://www.ncbi.nlm.nih.gov/geo/) database.

### 2.2 Weighted gene co‐expression network analysis

Weighted gene co‐expression network analysis (WGCNA) was conducted on the RNA‐seq data of 77 ACC samples and 128 normal adrenal gland samples using R package “WGCNA” (version 1.70‐3) according to the previously described standard method.^20^ One normal adrenal gland sample outlier was removed according to hierarchical clustering, and an experiential power value of 6 was used during co‐expression network construction.

### 2.3 Functional enrichment analysis

Gene ontology (GO) functional enrichment analysis was conducted using Metascape online tools (https://metascape.org, version v3.5.20240101). Gene set enrichment analysis (GSEA) was conducted using R package “clusterProfiler” (version 4.2.2) with parameters minGSSize = 15, maxGSSize = 500, nPermSimple = 10 000. The functional annotation file for GSEA was acquired from MsigDB (https://www.gsea‐msigdb.org/gsea/). PPI network analysis was conducted by STRING online tools (https://cn.string‐db.org/).

### 2.4 Human formalin‐fixed paraffin‐embedded samples

Fifteen formalin‐fixed paraffin‐embedded (FFPE) samples of ACC, 13 paired FFPE samples of adrenocortical adenoma, and corresponding tumour‐adjacent normal adrenal cortical tissue were acquired from the Department of Pathology, Shandong Provincial Hospital Affiliated to Shandong First Medical University in Jinan from 2013 to 2023. None of the patients underwent adjuvant therapy prior to surgery. The detailed clinicopathological features of the 15 ACC patients are provided in Table S1.

### 2.5 Plasmid, siRNA, and lentivirus construction

The pcDNA3.1‐*FGL1* plasmid was constructed by Genecefe Biotechnology Co., Ltd. Plasmid DNA was purified using the Endofree Plasmid Kit (QIAGEN). Two small interfering RNAs (siRNAs) targeting *CENPM* (si*CENPM*‐1, si*CENPM*‐2) and negative control (si*NC*) were synthesized by GenePharma Co., Ltd. Recombinant LV‐luciferase‐sh*CENPM*‐Puro and LV‐luciferase‐sh*Scramble*‐Puro lentiviruses were constructed by GenePharma Co., Ltd. The siRNA and shRNA sequences are shown in Table S2.

### 2.6 Plasmid, siRNA transfections and lentivirus infection

The human ACC cell lines, NCI‐H295R (RRID: CVCL_0458) and SW‐13(RRID: CVCL_0542) were kindly provided by Cell Bank, Chinese Academy of Sciences. H295R cells were cultured in DMEM/F12 medium supplemented with 10% fetal bovine serum (FBS), and .5% solution of insulin‐transferrin‐selenium. SW‐13 cells were cultured in Leibovitz's L‐15 medium containing 10% FBS. The recombinant plasmid was transfected into SW‐13 cells using PolyFast Transfection Reagent (MCE). siRNAs were transfected into H295R or SW‐13 cells with GP‐transfect‐Mate kit (GenePharma). Recombinant lentiviruses were used to infect the SW‐13 cells. Briefly, SW‐13 cells were grown in six‐well plates at a density of 2.0 × 10^5^ cells per well. When the cells were 50% confluent, they were infected with recombinant lentiviruses in the presence of 5 µg/mL polybrene (GenePharma, Co., Ltd.) at an MOI of 20. Stable clones were selected using 1.0 µg/ml puromycin for 10–14 days.

### 2.7 Immunohistochemistry and immunofluorescence staining

Human and animal FFPE tissue sections were stained with primary antibodies against IgG, CENPM, or ki67 at 4°C overnight. The Corresponding HRP‐labeled secondary antibodies were used for 1 h at room temperature (RT). The average positive CENPM signal was evaluated for five randomly selected regions in each section.

Tissue sections and cell slides were stained with primary antibodies against IgG, CENPM, COL2A1, or FGL1 overnight at 4°C, and subsequently incubated with FITC‐ or Cy3‐conjugated secondary antibodies. Cell nuclei were shown by DAPI staining. Images were captured using a fluorescence microscope (Leica; Olympus). The density of positive cells was evaluated in five randomly selected fields. The antibodies used in this study are listed in Table S3.

### 2.8 Real‐time quantitative PCR

Total cellular RNA was isolated using the Trizol reagent. CDNA was obtained by reverse transcription using a cDNA synthesis kit (Toyobo). Real‐time quantitative PCR was carried out using SYBR Green Realtime PCR Master Mix (Toyobo) on a QuantStudio 5 Real‐Time PCR System (Thermofisher) following the manufacturer's protocol. The expression level of *CENPM* in the cell lines was measured using 2^−ΔΔCT^. *GAPDH* was used as a control. Primers of *CENPM* and *GAPDH* are listed in Table S2.

### 2.9 Western blot

Total proteins were extracted using RIPA lysis buffer with inhibitor cocktails and quantified using the BCA protein assay kit. Proteins were separated by SDS‐PAGE gel electrophoresis followed by electroblotting onto PVDF membranes. The membranes were incubated with primary antibodies against CENPM or FGL1 at 4°C overnight, and subsequently with corresponding HRP‐conjugated secondary antibodies for 1 h at RT. Protein signals were detected using the Gelview 1500 pro (BLT). The antibodies used in this study are listed in Table S3.

### 2.10 Cell proliferation, migration, and invasion assay

Cell proliferation was detected using a colony formation assay. After siRNA transfection for 72 h, 1000 cells were seeded in each well of a six‐well plate and cultured for approximately 20 days. The colonies were stained with a crystal violet solution. Cell migration was assessed using a wound‐healing assay. After 72 h of siRNA transfection, cells were incubated in PBS for 10 min, scratched the wound using a 100 µL pipette tip, and cultured in a medium with 2% FBS for 72 h. Cell invasion was assessed using a transwell invasion assay. Transfected cells were placed in the upper layer of the Matrigel chamber in a serum‐free medium, and a culture medium with 25% FBS was placed in the lower chamber.

### 2.11 DIA quantitative proteomics and bioinformatics analysis

SW‐13 cells transfected with si*NC* or si*CENPM*‐1 for 72 h were collected for DIA quantitative proteomic analysis. LC‐MS/MS high‐resolution mass spectrometry detection was performed in oebiotech Co., Ltd. using TimsTOF Pro (Bruker) and UltiMate 3000 (Thermo Fisher Scientific) systems. The mass spectrometry proteomics data have been deposited to the ProteomeXchange Consortium (https://proteomecentral.proteomexchange.org) via the iProX partner repositor^21^, ^22^ with the dataset identifier PXD058018.

### 2.12 Co‐immunoprecipitation assay

Total proteins from SW‐13 cells were extracted using IP lysis buffer and then incubated with FGL1 antibody or IgG overnight at 4°C under gentle agitation. The protein–antibody complex solution was incubated with protein A/G agarose beads for 4 h at RT, and the unbound proteins were washed away. The captured proteins were separated from the beads using elution buffer and were used for western blot analysis of CENPM protein expression. The antibodies used in this study are listed in Table S3.

### 2.13 Metastatic ACC xenograft mouse model

Six male NPG (NOD.Cg‐Prkdc^scid^ Il2rg^tm1Vst^/Vst) mice were bought from Vitalstar Biotechnology Co., Ltd. and divided equally into two groups: LV‐sh*CENPM* and negative control. Each mouse was intravenously injected via the caudal vein with 2 × 10^6^ SW‐13 cells stably transfected with LV‐luciferase‐sh*CENPM*‐Puro or LV‐luciferase‐sh*Scramble*‐Puro. Twenty‐eight days after injection, the mice were intraperitoneally injected with d‐luciferin sodium salt (Meilunbio) and imaged by the Small Animal In Vivo Imaging System (PerkinElmer). Ethical approval for this study was granted by the Animal Research Ethics Committee of Shandong Second Medical University.

### 2.14 Statistical analysis

Data were analyzed using GraphPad Prism software (version 9.0) and were presented as mean ± standard deviation (SD). For those with more than two groups, analysis of variance (ANOVA) was performed to ensure *p* < .05. Differences between the two groups were analyzed using Student's *t*‐test, *p* < .05. *p < .05, **p < .01, ***p < .001, ****p < .0001.

## 3 RESULTS

### 3.1 Identification of gene module correlated with ACC metastasis

**FIGURE 1:**
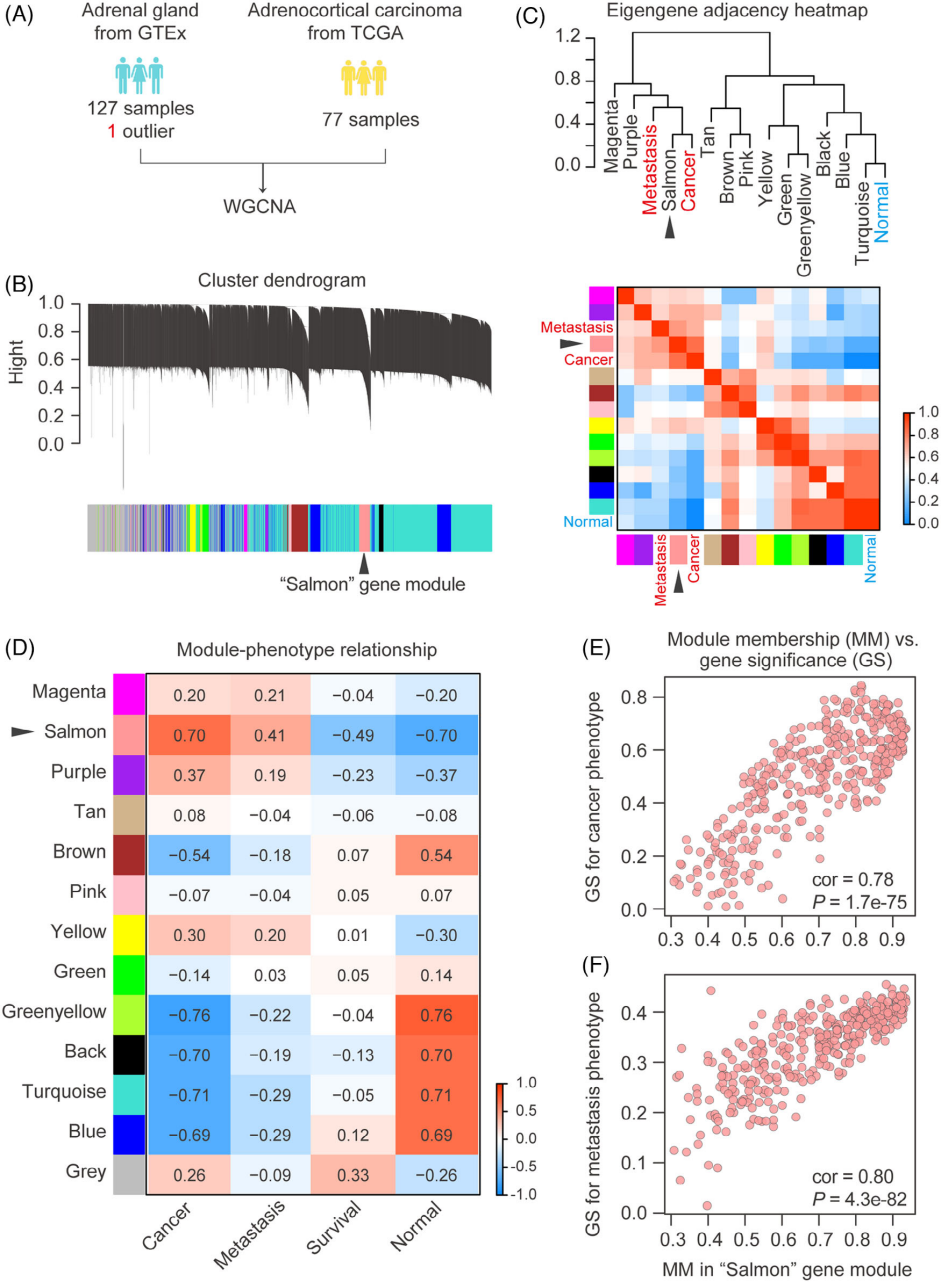
Screening of ACC metastasis‐related genes by WGCNA. (A) Sources of RNA‐seq data. (B) Gene dendrogram obtained by average linkage hierarchical clustering. (C) Hierarchical clustering dendrogram (top), adjacency heatmap of module eigengenes and sample phenotypes (bottom). The “Salmon” gene module had a high degree of adjacency to “Cancer” and “Metastasis”. (D) Pearson correlation of gene modules with clinical phenotypes including cancer, normal, metastasis and survival. (E) Scatterplot of module membership in “Salmon” module and gene significance (GS) for cancer phenotype. The “Salmon” module had a high correlation with the “Cancer” phenotype. (F) Scatterplot of module membership in the “Salmon” module and gene significance for metastasis phenotype. The “Salmon” module had a high correlation with the “Metastasis” phenotype.

To characterize the potential regulatory genes involved in the progression and metastasis of ACC, we performed WGCNA on ACC samples and normal adrenal gland samples (Figure 1A). All expressed genes were initially filtered using the median absolute deviation method, resulting in a total of 12 035 genes for co‐expression network construction. Subsequent hierarchical clustering analysis categorized these genes into 12 distinct gene modules, each assigned a unique colour for identification, while unclustered genes were labelled as grey (Figure 1B). Eigengene adjacency analysis showed the “Salmon” gene module, comprising 363 genes, exhibited the highest eigengene adjacency to both “Cancer” and “Metastasis” phenotypes, and the lowest eigengene adjacency to “Normal” phenotypes (Figure 1C). Consistently, Pearson correlation analysis revealed that the “Salmon” module showed a positive correlation with “Cancer” (cor = .70), and “Metastasis” (cor = .41), as well as a negative correlation with “Survival” (cor = –.49), and “Normal” (cor = –.70) (Figure 1D). Additionally, the module membership (MM) of genes in the “Salmon” module was significantly correlated with their gene significance (GS) for the “Cancer” (cor = .78, *p *< .01) and “Metastasis” (cor = .80, *p *< .01) phenotypes (Figure 1E,F). Collectively, our WGCNA results revealed that the “Salmon” gene module was closely associated with the tumorigenesis and metastasis of ACC.

### 3.2 CENPM was the hub gene related to ACC metastasis

**FIGURE 2:**
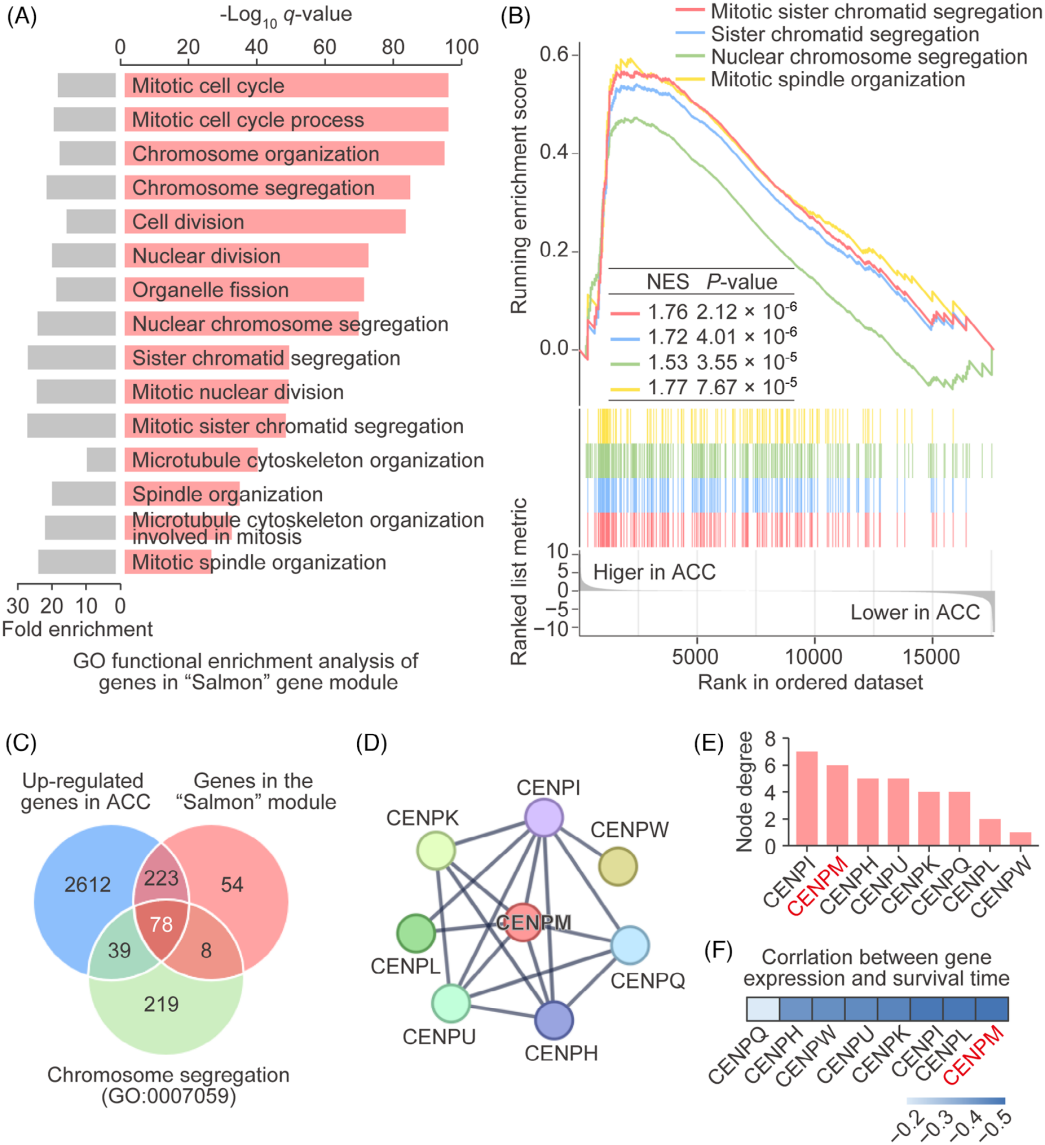
Identification of hub genes related to ACC metastasis. (A) GO functional enrichment analysis of genes in the “Salmon” module. Both fold enrichment (left, grey) and −log10p‐value (right, salmon) were shown. (B) Gene set enrichment analysis. (C) Venn diagram of genes in the “Salmon” module, upregulated genes in ACC and genes involved in chromosome segregation (GO:0007059). (D) PPI networks based on the 78 genes. The largest PPI network was comprised of eight CENP family members. Genes with node degree less than 1 were not included. (E) Node degree of the eight CENP family members in the above PPI network. CENPM had the second‐highest node degree. (F) Spearman correlation of eight CENP family members with ACC overall survival. *CENPM* showed the highest negative correlation with ACC overall survival.

To further identify the key genes driving ACC metastasis, we conducted a functional enrichment analysis of genes in the “Salmon” module. GO enrichment analysis revealed that the genes in the “Salmon” module were predominantly involved in mitotic cell cycle‐related processes, particularly in terms connected with chromosome segregation and spindle organization (Figure 2A). Additionally, GSEA showed that gene sets involving “Mitotic sister chromatid segregation” (NES = 1.76, *p *< .01), “Sister chromatid segregation” (NES = 1.72, *p *< .01), “Nuclear chromosome segregation” (NES = 1.53, *p *< .01), and “Mitotic spindle organization” (NES = 1.77, *p *< .01) pathways were significantly upregulated in ACC (Figure 2B).

To identify the genes within the “Salmon” module that involved the regulation of chromosome segregation in ACC, we overlapped the “Salmon” module genes with ACC upregulated genes and chromosome segregation‐related genes. This analysis identified 78 genes within the “Salmon” module that were potentially related to the regulation of chromosome segregation in the ACC (Figure 2C). To further pinpoint hub genes, protein–protein interaction (PPI) network was constructed based on the 78 genes we identified. Notably, the largest PPI network was comprised of eight CENP family members (CENPI, CENPM, CENPH, CENPU, CENPK, CENPQ, CENPL, and CENPW; Figure 2D). We noticed that CENPM had the second‐highest node degree (Figure 2E) and the strongest negative correlation with ACC patient overall survival time (Figure 2F), suggesting that CENPM was the hub gene related to ACC metastasis.

### 3.3 CENPM was upregulated in ACC, and associated with metastasis and poor prognosis of ACC patients

**FIGURE 3:**
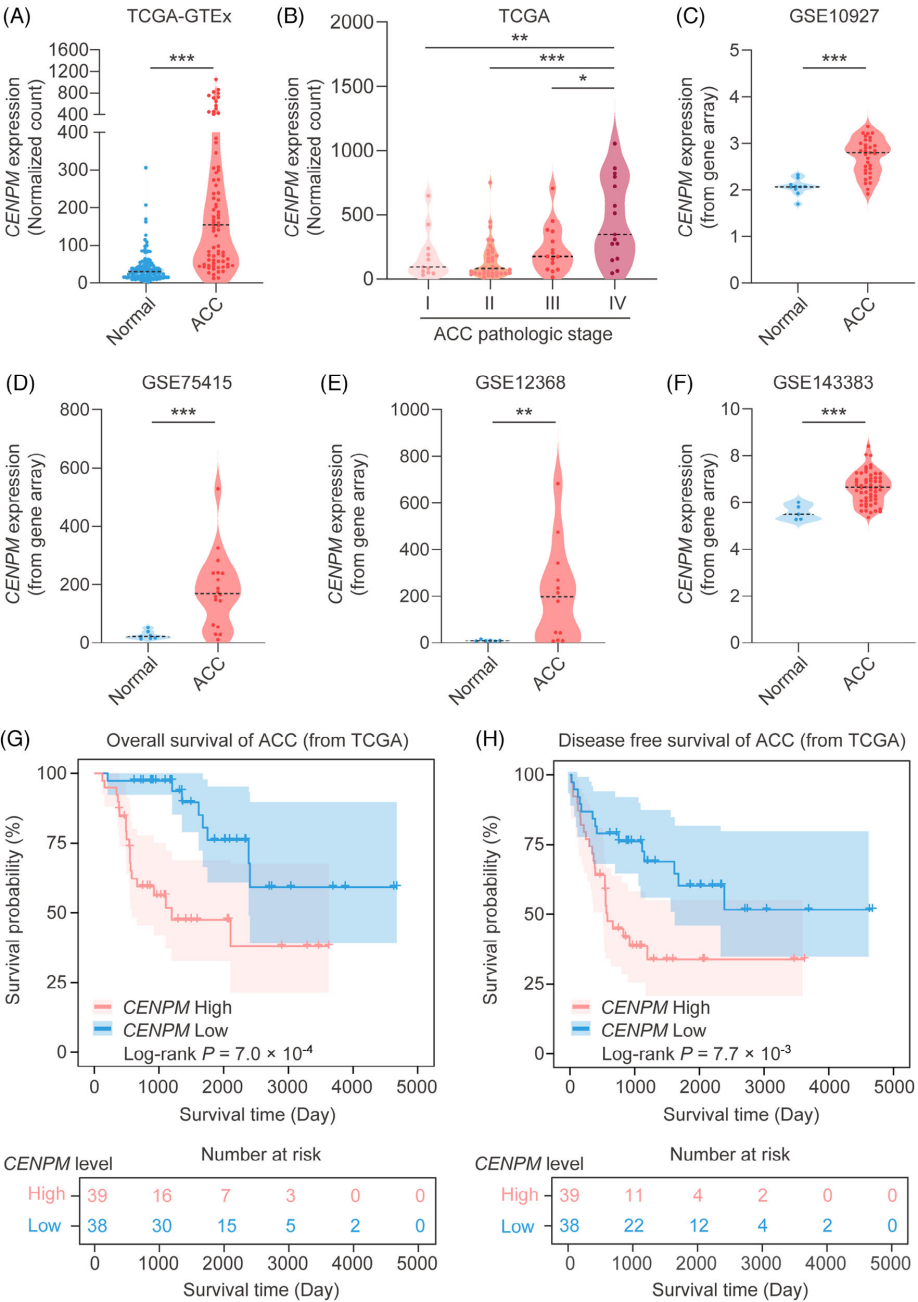
Expression and survival analysis of CENPM in ACC patients. (A) CENPM mRNA expression of normal adrenal gland (from GTEx) and ACC (from TCGA). (B) CENPM mRNA expression in ACC with different pathologic stages (TNM stage). (C–F) The mRNA expression of CENPM in four ACC GEO datasets (GSE10927, GSE75415, GSE12368, GSE143383). (G) Kaplan–Meier survival analysis of OS in ACC. (H) Kaplan–Meier survival analysis of DFS in ACC. The samples were assigned into CENPM high/low cohorts by the median value. For those with more than two groups, ANOVA analysis was performed to ensure *p* < .05, and then the Student's *t*‐test was performed between the two groups. *p < .05, **p < .01, ***p < .001.

The RNA‐seq data in TCGA and GTEx databases showed that the mRNA levels of *CENPM* were significantly elevated in ACC samples compared with normal adrenal gland samples (Figure 3A). Furthermore, ACC patients in stage IV displayed higher levels of *CENPM* than those in stage I, stage II, and stage III (Figure 3B), indicating the mRNA expression of *CENPM* was correlated with ACC metastasis. The microarray data in GSE10927 (Figure 3C), GSE75415 (Figure 3D), GSE12368 (Figure 3E), and GSE143383 (Figure 3F) also indicated that *CENPM* mRNA levels were highly upregulated in ACC patients.

**FIGURE 4:**
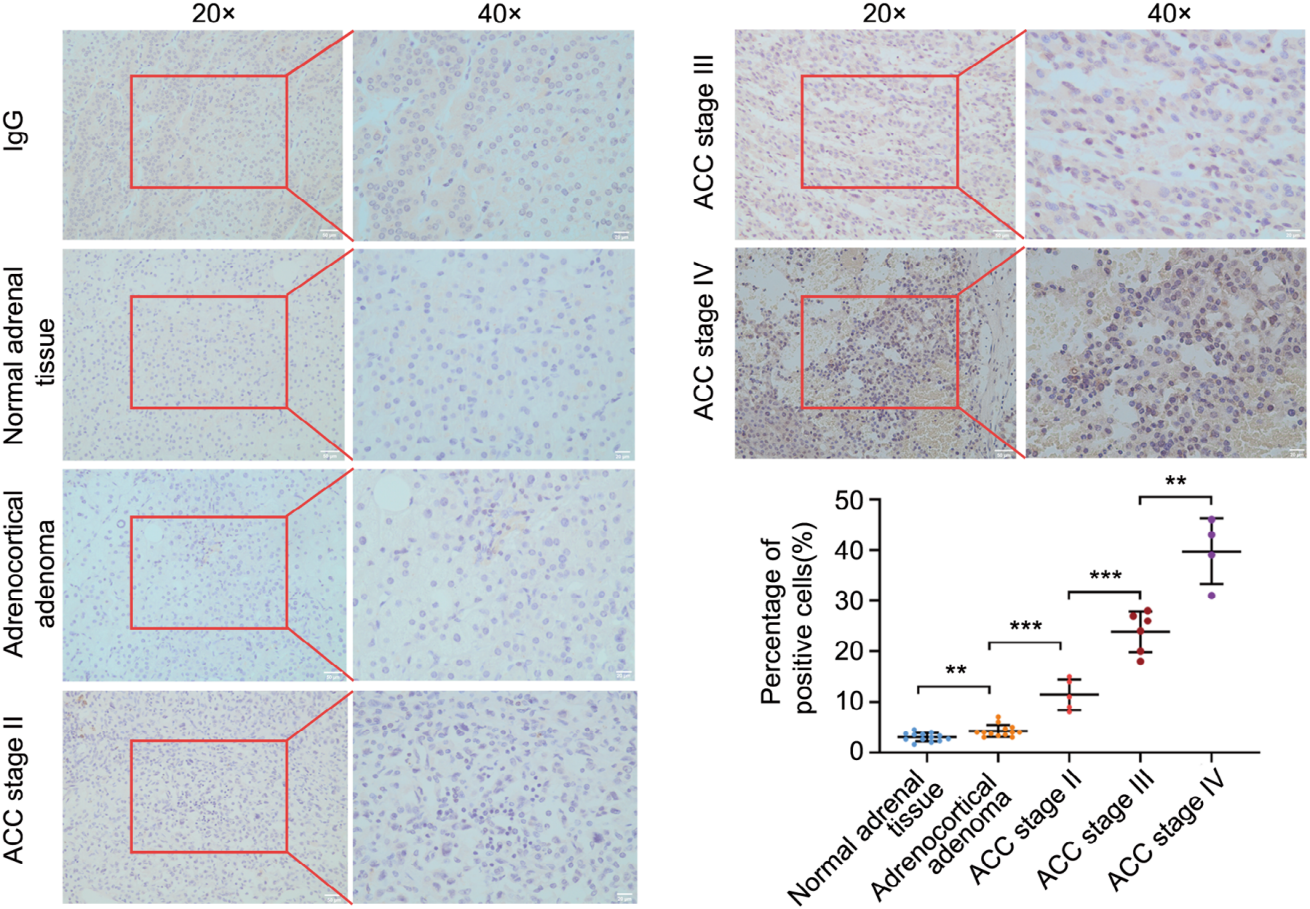
Immunochemistry of CENPM protein expression in ACC. FFPE sections of normal adrenal tissue, adrenocortical adenoma, ACC stage II, ACC stage III, and ACC stage IV (ENSAT stage) were stained. IgG was used as a negative control. Images were shown at 20× and 40× magnification. ANOVA analysis was performed to ensure *p* < .05, and then the Student's *t*‐test was performed between the two groups. **p < .01, ***p < .001.

The immunohistochemistry results revealed that the protein level of CENPM was enhanced in ACC patients than in normal adrenal gland tissues and adrenocortical adenoma patients. In addition, ACC patients in stage IV displayed higher levels of CENPM than those in stage II and stage III (Figure 4), suggesting that the protein expression of CENPM was also correlated with ACC metastasis.

To further elucidate the relationship between *CENPM* and ACC progression, survival analysis was conducted on ACC patients grouped by higher and lower CENPM expression levels. As shown by the Kaplan–Meier curves for OS and DFS, ACC patients with high *CENPM* levels displayed poorer overall survival (Figure 3G) and disease‐free survival (Figure 3H) than those with low levels of *CENPM*, providing evidence that the expression of *CENPM* in ACC negatively correlated with the survival of patients.

### 3.4 Knockdown of CENPM inhibited the proliferation, migration, and invasion of ACC cells

**FIGURE 5:**
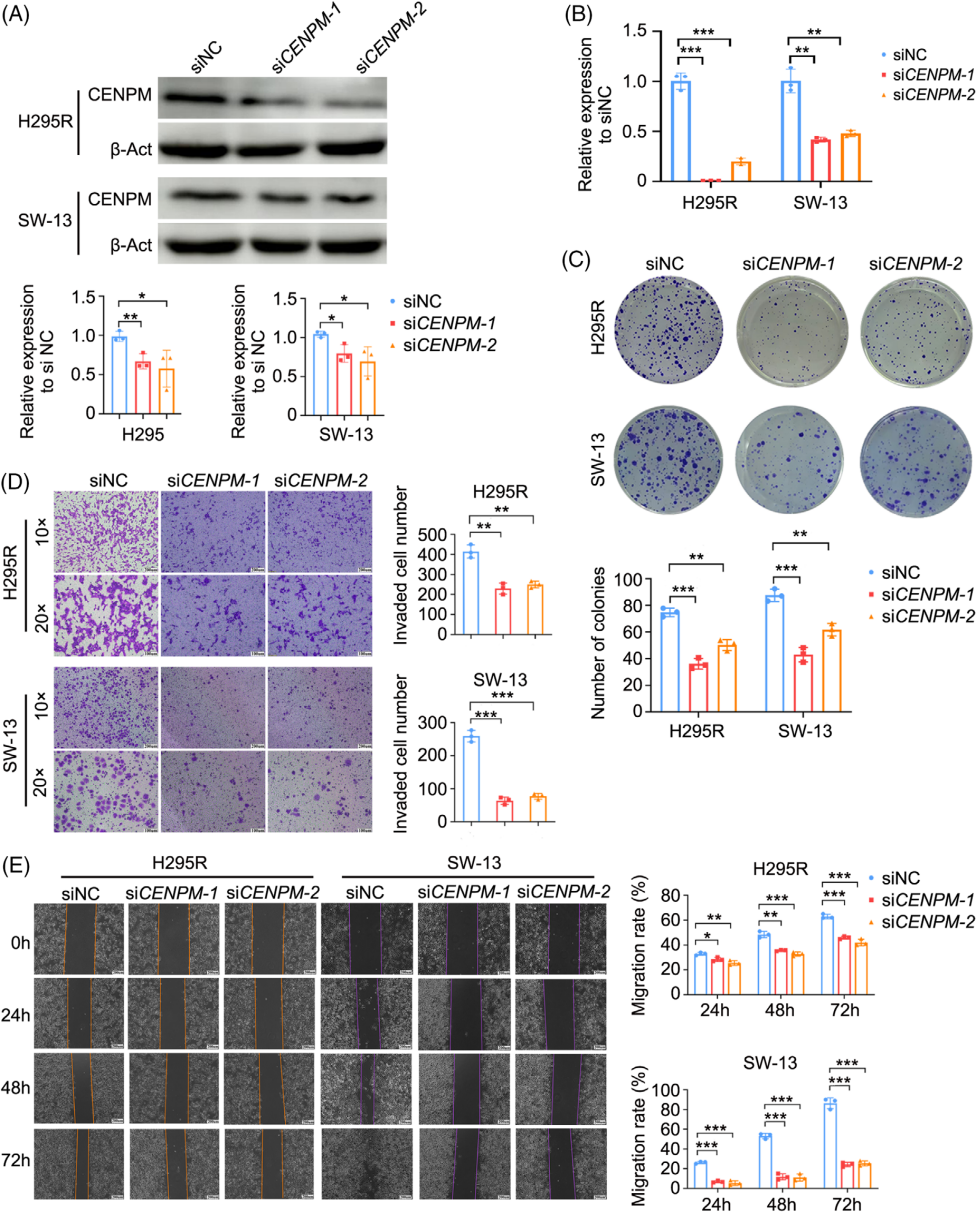
The impact of CENPM knockdown on the proliferation, migration and invasion of ACC cells in vitro. (A) Western blot analysis of CENPM protein in both H295R and SW‐13 cells transfected with siCENPM‐1 or siCENPM‐2 after 72 h. (B) Real‐time quantitative PCR analysis of CENPM mRNA expression in both H295R and SW‐13 cells transfected with siCENPM‐1 or siCENPM‐2 after 48 h. (C) Colony formation assay. (D) Transwell invasion assay. (E) Wound healing assay (scale bar = 200 µm). ANOVA analysis was performed to ensure *p* < .05, and then Student's *t*‐test was performed between two groups. *p < .05, **p < .01, ***p < .001.

To verify the impact of CENPM on ACC metastasis, we silenced the expression of *CENPM* by transfecting siRNA into two ACC cell lines H295R and SW‐13. The mRNA and protein levels of *CENPM* were significantly downregulated after siRNA transfection (Figure 5A,B). Silencing of *CENPM* led to inhibition of proliferation in both H295R and SW‐13 cells as shown by the colony formation assay (Figure 5C). Wound healing and transwell assays revealed that migration and invasion decreased in *CENPM* knockdown ACC cells (Figure 5D,E).

### 3.5 Knockdown of CENPM inhibited collagen‐containing extracellular matrix signalling

**FIGURE 6:**
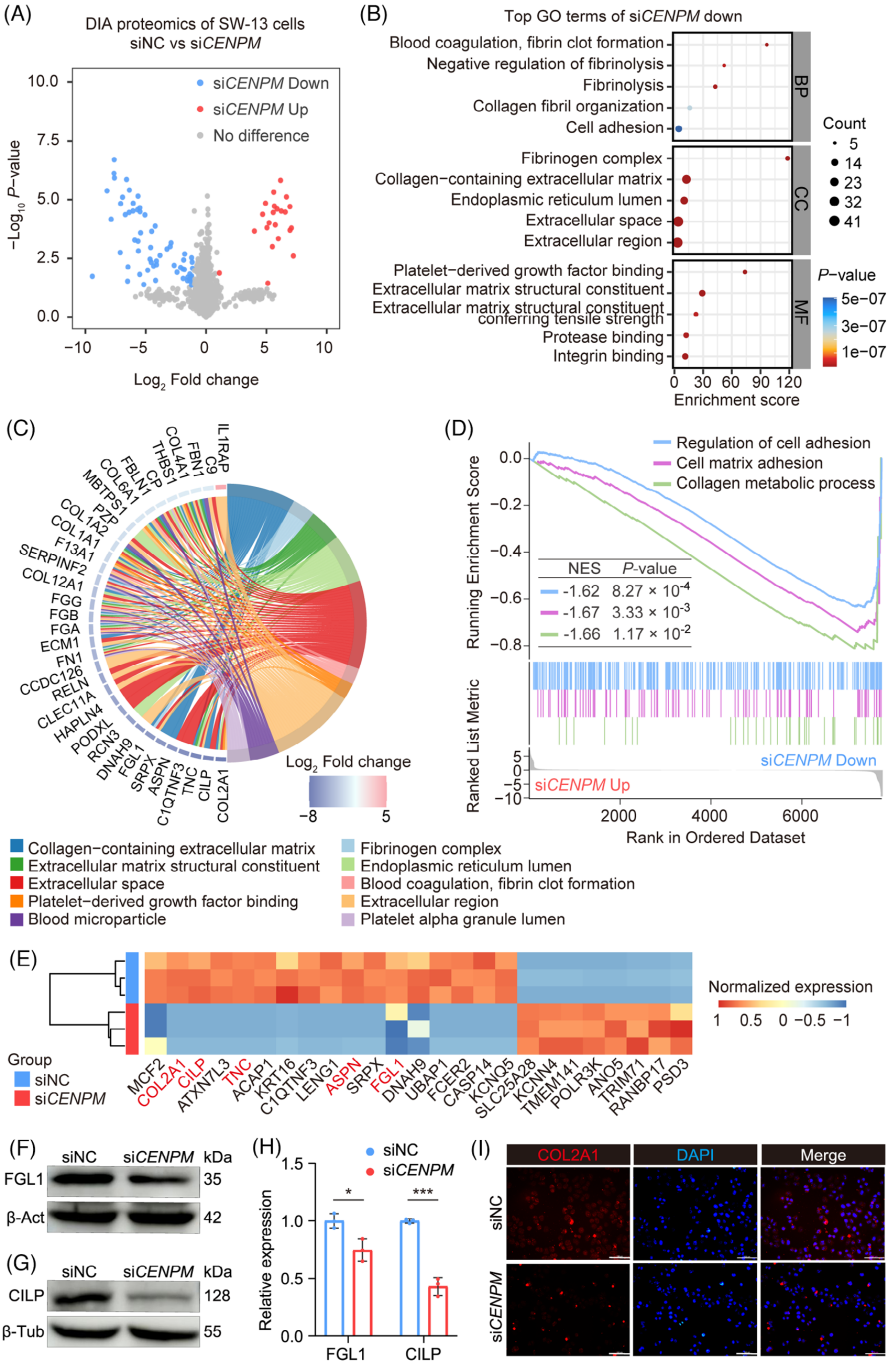
DIA quantitative proteomics analysis of CENPM knockdown SW‐13 cells. (A) Volcano plot of DIA quantitative proteomics. (B) GO functional enrichment analysis of 62 downregulated proteins. The top biological process (BP), cellular component (CC), and molecular function (MF) were shown by bubble chart. (C) Chord diagram of the 62 downregulated proteins. The downregulated proteins were primarily enriched in “collagen‐containing extracellular matrix” signalling. (D) GSEA of DIA quantitative proteomics. (E) Heatmap of proteins downregulated 64‐fold or more. Among them, COL2A1, CILP, TNC, ASPN, and FGL1 were in “collagen‐containing extracellular matrix” signalling (shown in red). (F) Western blot analysis of FGL1 in CENPM knockdown SW‐13 cells. β‐Actin was used as a negative control. (G) Western blot analysis of CILP in CENPM knockdown SW‐13 cells. β‐Tubulin was used as a negative control. (H) Statistical analysis of FGL1 and CILP protein expression. (I) Immunofluorescence of COL2A1 in *CENPM* knockdown SW‐13 cells (scale bar = 100 µm). Student's *t*‐test was performed between two groups. *p < .05, ***p < .001.

We further conducted DIA quantitative proteomics to identify downstream effectors following *CENPM* knockdown. The knockdown of *CENPM* in ACC cells resulted in 24 upregulated proteins and 62 downregulated proteins using a twofold cut‐off threshold (Figure 6A).

Unexpectedly, GO enrichment analysis revealed that the downregulated proteins following CENPM knockdown were enriched in biological processes such as collagen fibril organization and cell adhesion (Figure 6B,C). Additionally, GO cellular component analysis indicated that these proteins were predominantly localized in the collagen‐containing extracellular matrix and extracellular space (Figure 6B,C). Furthermore, GSEA confirmed that the expression of genes related to cell‐matrix adhesion and collagen metabolism was significantly downregulated upon CENPM silencing (Figure 6D). Collectively, these findings suggest that CENPM serves as a positive regulator of collagen‐related pathways, with its knockdown leading to the dysregulation of collagen organization and cell adhesion in ACC.

Of the proteins downregulated 2^6^‐fold or more, COL2A1, CILP, TNC, ASPN, and FGL1 were involved in “collagen‐containing extracellular matrix” signalling (Figure 6E). The protein levels of FGL1 and CILP were validated in *CENPM* knockdown SW‐13 cells by western blot analysis (Figure 6F–H) and the protein levels of COL2A1 were validated in *CENPM* knockdown SW‐13 cells by immunofluorescence (Figure 6I). The results suggested that the protein levels of FGL1, CILP, and COL2A1 were downregulated in *CENPM* knockdown cells, which was consistent with the results of DIA quantitative proteomics.

### 3.6 CENPM interacted with FGL1 to regulate migration and invasion of ACC cells

**FIGURE 7:**
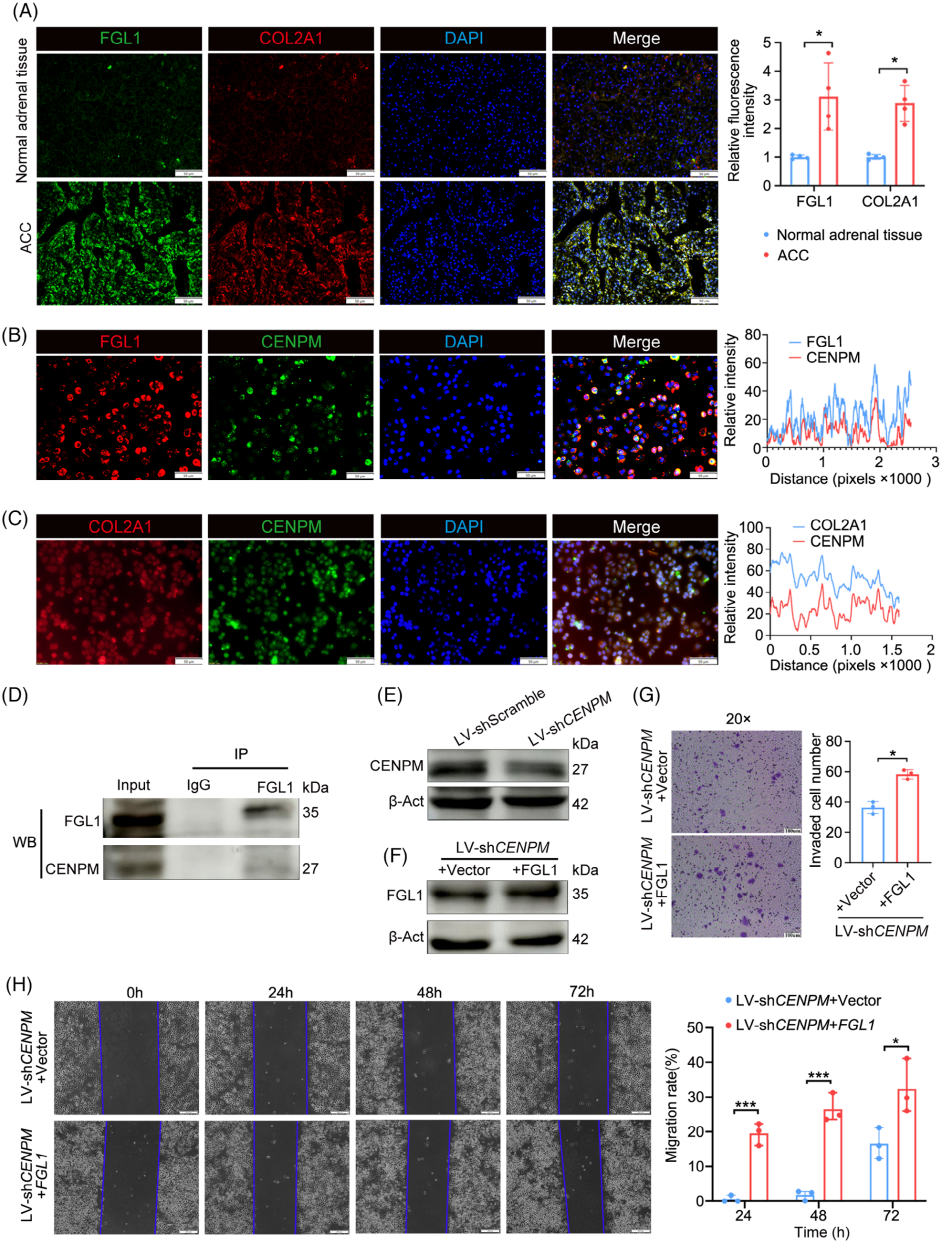
Identification of downstream target of CENPM. (A) Immunofluorescence of FGL1 and COL2A1 in FFPE sections of normal adrenal tissues (n = 4) and advanced ACC patients (n = 4) (scale bar = 50 µm). (B) Immunofluorescence of FGL1 and CENPM in SW‐13 cells (scale bar = 50 µm). (C) Immunofluorescence of COL2A1 and CENPM in SW‐13 cells (scale bar = 50 µm). (D) Co‐immunoprecipitation assay in SW‐13 cells. Cell lysates were incubated with FGL1 antibody. IgG was used as a negative control. Western blot analysis of CENPM protein expression was performed. (E) Western blot analysis of CENPM in SW‐13 cells stably infected with LV‐shCENPM. (F) Western blot analysis of FGL1 in LV‐shCENPM SW‐13 cells transfected with pCDNA3.1‐FGL1. (G) Transwell invasion assay of LV‐shCENPM SW‐13 cells transfected with pCDNA3.1‐FGL1 (scale bar = 100 µm). (H) Wound healing assay of LV‐shCENPM SW‐13 cells transfected with pCDNA3.1‐FGL1 (scale bar = 200 µm). ANOVA analysis was performed to ensure *p* < .05, and then Student's t‐test was performed between two groups. *p < .05, ***p < .001.

The protein levels of COL2A1 and FGL1 were detected in FFPE sections of ACC and normal adrenal glands by immunofluorescence staining. In normal adrenal glands, COL2A1 and FGL1 proteins were in low levels and located in the extracellular matrix, whereas in ACC patients, COL2A1 and FGL1 proteins were in high levels and located in the cytoplasm and nucleus (Figure 7A). The co‐expression of FGL1 and CENPM proteins was remarkable, whereas the co‐expression of COL2A1 and CENPM proteins was not obvious (Figure 7B,C). Furthermore, the co‐immunoprecipitation assay showed the physical interaction between CENPM and FGL1 (Figure 7D).

To determine whether CENPM regulates the migration and invasion of ACC cells via interacting with FGL1, a rescue experiment was performed. SW‐13 cells stably infected with recombinant LV‐luciferase‐sh*CENPM*‐Puro virus (Figure 7E) were transfected with vector (negative control) or pcDNA3.1‐*FGL1* plasmids (Figure 7F). Wound healing and transwell invasion assays were performed. Overexpression of FGL1 promoted the migration and invasion of *CENPM* knockdown ACC cells (Figure 7G,H). These results demonstrated that CENPM regulated the migration and invasion of ACC cells via FGL1.

### 3.7 Knockdown of CENPM suppressed the liver metastasis of ACC in vivo

**FIGURE 8:**
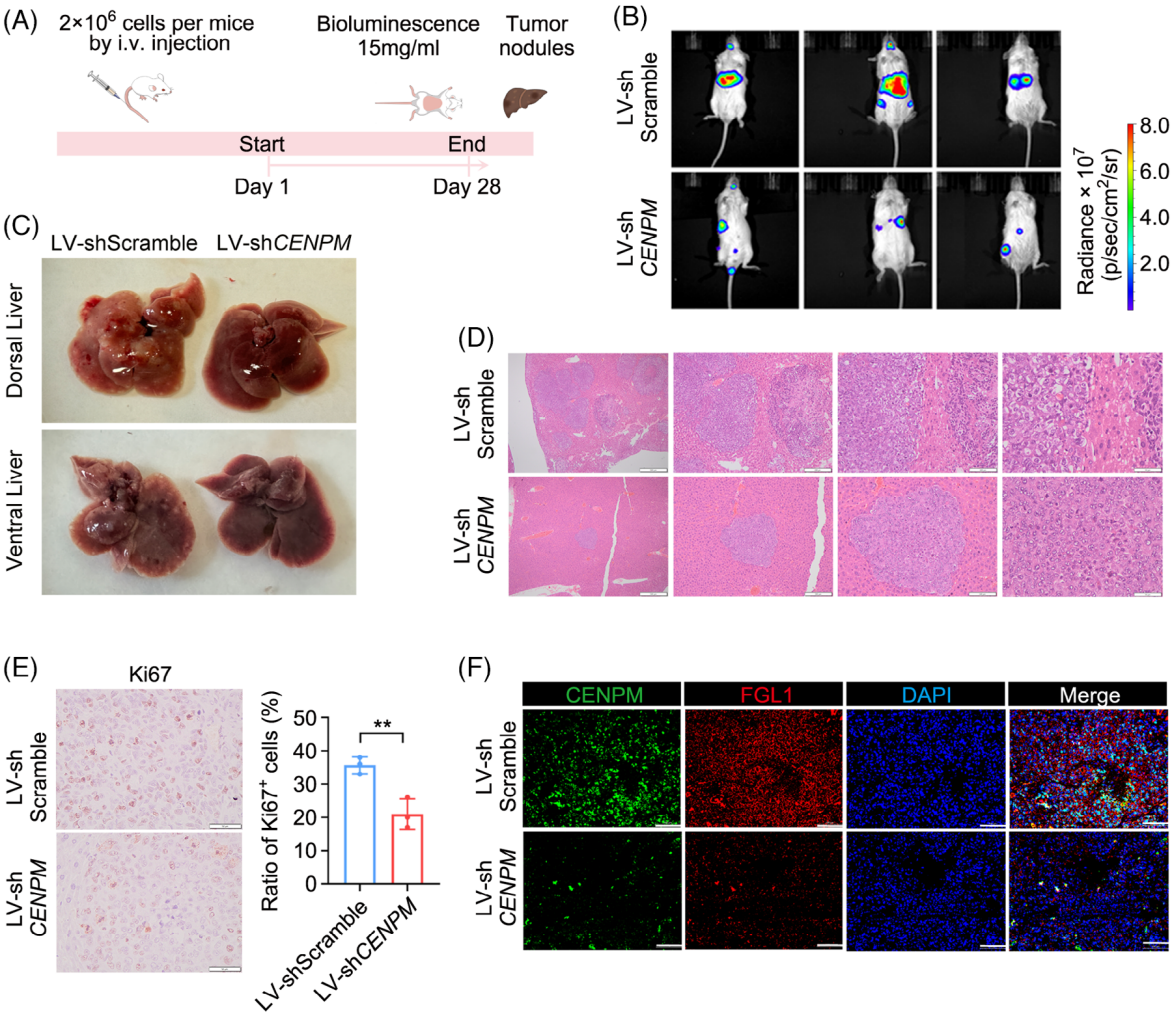
The impact of CENPM knockdown on liver metastasis of ACC in vivo. (A) A schematic diagram of metastatic ACC xenograft mouse model. (B) Bioluminescence imaging of metastatic ACC xenograft mice on Day 28. (C) Macrograph of liver metastatic ACC tumours. (D) HE staining of liver metastatic ACC tumours. From left to right, images were shown at 4×, 10×, 20×, and 40× magnification. (E) ki67 staining of metastatic ACC tumours. (F) Immunofluorescence of CENPM and FGL1 in metastatic ACC tumors. Student's *t*‐test was performed between two groups. **p < .01.

A metastatic ACC xenograft mouse model was developed by injecting SW‐13 cells stably infected with LV‐luciferase‐sh*CENPM*‐Puro or LV‐luciferase‐sh*Scramble*‐Puro (normal control) into NPG mice via a caudal vein (Figure 8A). Normal controls showed strong bioluminescence signals in the upper right quadrant of the abdomen, whereas weak or no signals were observed in the upper right quadrant of the abdomen in mice injected with LV‐sh*CENPM* ACC cells on day 28 (Figure 8B). A large number of white tumour nodules were observed in the livers of normal controls. In contrast, there were fewer white tumour nodules in the livers of mice that received LV‐sh*CENPM* ACC cells (Figure 8C). No visible tumour nodules were observed in the lung, spleen, and kidney, suggesting that the liver was the primary metastatic target for ACC cells. HE staining of liver specimens showed much fewer ACC metastatic tumour nodules in mice injected with LV‐sh*CENPM* ACC cells than in normal controls (Figure 8D). Knockdown of *CENPM* also resulted in a decrease of ki67 positive cells and a decline of FGL1 levels, as shown by ki67 staining and immunofluorescence of ACC metastatic tumour nodules, respectively (Figure 8E,F).

## 4 DISCUSSION

CENPM is a key CCAN member essential for accurate chromosome segregation during cell division. Ablation of CENPM causes failure in chromosome alignment and induces mitotic arrest.^12^, ^23^ CENPM is upregulated in several cancers such as melanoma,^24^ hepatocellular carcinoma,^25^ pancreatic cancer,^26^ lung adenocarcinoma,^27^ and ovarian cancer.^28^ Upregulation of CENPM promotes hepatocarcinogenesis and facilitates tumour metastasis of melanoma, pancreatic cancer, and lung adenocarcinoma.^24^, ^25^, ^26^, ^27^ In our study, CENPM was found to be upregulated in ACC, correlated with metastasis and poor prognosis of ACC, and functioned as a key gene in driving ACC metastasis.

It is well known that precise prediction of prognosis helps risk stratification and personalized therapeutic strategy. Several available pathological and molecular prognostic factors are used to predict risk assessment; however, tumour stage remains the strongest prognostic factor.^3^, ^29^ Although the survival for metastatic ACC is dismal, there are some long‐term survivors, suggesting the heterogeneity of metastatic ACC and the necessity of an accurate prognostic indicator.^30^ CENPM may account for the heterogeneity of metastatic ACC, and may be used as a superior prognostic indicator.

Many efforts have been made to discover the driving force of ACC metastasis and explore new therapeutic targets.^29^, ^31^, ^32^ Inactivation of p53 and activation of β‐catenin induces metastatic ACC.^33^, ^34^, ^35^ β‐catenin activation is significantly associated with more frequent mitoses and a higher Weiss score.^36^ In our study, CENPM was found the key driving force of ACC metastasis. Therefore, CENPM might be a putative therapeutic target for ACC metastasis.

In our study, we also found that the knockdown of *CENPM* inhibited collagen‐containing extracellular matrix (ECM) signalling. In normal tissues and organs, the ECM forms a scaffold and a barrier. However, the constituents and architecture of ECM can be remodelled by cancer cells with fibrillar collagen production and alignment. Fibrotic, stiffened tumour stroma fosters favourable tracks for local invasion and subsequent dissemination of tumour cells.^37^, ^38^, ^39^ Nevertheless, collagens are also expressed on tumour cells and can trigger immune inhibitory signalling through leukocyte‐associated Ig‐like receptor‐1,^40^ which may explain our findings that, in normal adrenal glands, COL2A1 and FGL1 proteins were in low levels and located in the extracellular matrix, whereas in ACC patients, COL2A1 and FGL1 proteins were in high levels and located in cytoplasm and nucleus.

FGL1, also called fibrinogen‐like protein 1, is a member of the fibrinogen‐associated protein family and is the ligand of immune checkpoint LAG3. By binding with LAG3, FGL1 inhibits T cell activation.^41^, ^42^ FGL1 is confined to the liver and pancreas under normal conditions, and elevated in human cancers such as lung cancer, prostate cancer, melanoma, and colorectal cancer, associated with a poor prognosis and resistance to anti‐PD‐1/B7‐H1 therapy.^42^, ^43^ FGL1 promotes metastatic tumour progression via mediating immune escape. Upregulation of FGL1 facilitates tumour progression and metastasis in liver cancer, non‐small‐cell lung cancer and esophageal squamous cell carcinoma.^44^, ^45^, ^46^, ^47^ Increasing studies have emphasized the potential of FGL1 as the next immune checkpoint target.^43^ In our study, FGL1 was overexpressed in ACC and promoted the metastasis of ACC. The interaction between CENPM and FGL1 indicates the involvement of immune suppression in CENPM‐mediated ACC metastasis, which needs further investigation in the future.

## AUTHOR CONTRIBUTIONS

Cunru Zou; Yu Zhang; and Chengyue Liu: Investigation; validation; formal analysis; and visualization. Yaxin Li and Congjie Lin: Investigation and data curation. Hao Chen: Software. Jiangping Hou and Guojun Gao: Methodology. Zheng Liu: Resources; data curation; and funding acquisition. Qiupeng Yan: Software; funding acquisition; formal analysis; visualization; and writing–review and editing. Wenxia Su: Funding acquisition; data curation; project administration; resource; and writing–original draft.

## CONFLICT OF INTEREST STATEMENT

The authors declare no conflict of interest.

## ETHICS STATEMENT

The animal study was approved by the Animal Research Ethics Committee of Shandong Second Medical University (issue no: 2024SDL618).

## CONSENT FOR PUBLICATION

All the authors have read and approved the publication of this manuscript.

## Supporting information



Supporting Information

Supporting Information

Supporting Information

## Data Availability

The RNA‐seq data are available in TCGA and GTEx databases. Microarray data are available in GSE10927, GSE75415, GSE12368, and GSE143383. DIA quantitative proteomics data are available in ProteomeXchange Consortium PXD058018 (https://proteomecentral.proteomexchange.org).
