# Supplementary material for: Identification of CENPM as a key gene driving adrenocortical carcinoma metastasis via physical interaction with immune checkpoint ligand FGL1
Source: Clin Transl Med. 2025 Jan 8;15(1):e70182. doi: 10.1002/ctm2.70182 (PMC11707433; doi:10.1002/ctm2.70182)
Supplement: Supplementary file 1 — Supporting Information [file CTM2-15-e70182-s003.docx]

**Table S1. The clinicopathological features of ACC patients**

| **Patients** | **Gender** | **Age** | **Diagnosis time**  **(year)** | **Tumor location** | **Tumor size** | **Metastasis** | **Tumor staging (ENSAT)** | **IHC** |
| --- | --- | --- | --- | --- | --- | --- | --- | --- |
| 1 | Female | 45 | 2013 | Right adrenal gland | 11×8×6 cm | No | Ⅱ | α-inhibin(+),CgA(-),Syn(+),MelanA(-),  S100(-), P53(-), Ki67+(40%) |
| 2 | Male | 24 | 2014 | Right adrenal gland | 14×11×8 cm | Liver | Ⅳ | CgA(+),Syn(+),MelanA(-), S100 (-), P53(-), Vimetnin(+), Heppar-1(-), CK(-), CK7(-), CK19(-), CD34(-), Ki67+ (60%) |
| 3 | Male | 52 | 2014 | Right adrenal gland | 6.5×6×1 cm | No | Ⅱ | α-inhibin((slight+),CgA(-),Syn(+),S100 (-),MelanA(-),Vimetnin(+), CK(-), P53+ (10%), Ki67+ (20%) |
| 4 | Male | 51 | 2015 | Right adrenal gland | 15×14×9 cm | Blood vessel | Ⅲ | α-inhibin(+), CgA(±), MelanA(+), S100 (-), HBM45(-), Ki67+(10%) |
| 5 | Female | 60 | 2016 | Right adrenal gland | 6×4×2 cm | Liver | Ⅳ | α-inhibin(+),CgA(-),Syn(+),S100(-), CK(+),Heppar(-),GPC-3(-),Arginase(-),  Ki67+ (15%) |
| 6 | Female | 33 | 2019 | Right adrenal gland | 6.5×5.8×3 cm | NO | Ⅱ | α-inhibin(+),CgA(-),Syn(+),MelanA(+), S100(-),Vimetnin(+),CK(+),CD56(+), CD10(slight+),CA9(-),Ki67+(15%) |
| 7 | Male | 52 | 2019 | Right adrenal gland | 5.5×4.5×3.5 cm | Kidney | Ⅲ | α-inhibin(+),CgA(-),Syn(+),MelanA(+), CD31(+),CK(+),PAX-8(-),Vimetnin(+), CK7(+),CD117(+),P504S(-),RCC(-),  CD10(-), Hepatocyte(-),Ki67+(20%) |
| 8 | Female | 19 | 2020 | Right adrenal gland | 12×11×8 cm | No | Ⅱ | α-inhibin(slight+), Syn(+), MelanA(-), S100 (-), CD56(+), CK(-), P53(-), Ki67+ (5%) |
| 9 | Female | 59 | 2021 | Right adrenal gland | 3×2.1×1 cm | Peripheral adipose tissue | Ⅲ | α-inhibin(+),CgA(-),Syn(+),MelanA(+), CK(-),Vimetnin(-),CA9(-),CD31(+),  Ki67+ (20%) |
| 10 | Male | 30 | 2021 | Right adrenal gland | 14×7×5 cm | Retroperitoneum | Ⅲ | α-inhibin(-),CgA(-),Syn(+),MelanA(+), S100(-),CK(-),PAX-8(-),HMB45(-), Calretinin(+), EMA(-),Ki67+(8%) |
| 11 | Female | 63 | 2021 | Retroperitoneum | 15×11×8 cm | Retroperitoneum | Ⅲ | α-inhibin(-),CgA(-),Syn(+),MelanA(+),  S-100(-),Vimetnin(+),CK(-),PHH3(-), Ki67+(25%) |
| 12 | Female | 52 | 2021 | left adrenal gland | 6×6×3.5 cm | Blood vessel | Ⅲ | α-inhibin(+),Syn(+),P53(+),CK(-),  Ki67+(30%) |
| 13 | Female | 55 | 2023 | left adrenal gland | 10×7×5 cm | No | Ⅱ | α-inhibin(-),CgA(-),Syn(+),MelanA(+), S100 (-),Vimetnin(-),CK(-), Ki67+(5%) |
| 14 | Female | 62 | 2022 | left adrenal gland | 7.6×6×5.8 cm | Liver | Ⅳ | α-inhibin(+), CgA(-), Syn(+), S100(-), NSE（partly+),CD15(-), Ki67+(5%) |
| 15 | Male | 51 | 2020 | Right adrenal gland | 8.4×6×5 cm | Liver | Ⅳ | α-inhibin(-),CgA(-),Syn(-),MelanA(-), S100 (-), Vimetnin(+), CK(+), Ki67+ (60%), EMA(+), NSE(-),Calretinin(-) |
