# Supplementary material for: Identification of CENPM as a key gene driving adrenocortical carcinoma metastasis via physical interaction with immune checkpoint ligand FGL1
Source: Clin Transl Med. 2025 Jan 8;15(1):e70182. doi: 10.1002/ctm2.70182 (PMC11707433; doi:10.1002/ctm2.70182)
Supplement: Supplementary file 2 — Supporting Information [file CTM2-15-e70182-s002.docx]

**Table S2. The sequences of siRNAs, shRNAs and primers**

| **siRNA/Primer** | **sense（5'-3'）** | **antisense（5'-3'）** |
| --- | --- | --- |
| si*NC* | UUCUCCGAACGUGUCACGUTT | ACGUGACACGUUCGGAGAATT |
| si*CENPM*-1 | GGAAGGCUUUAGGGCCACCTT | GGUGGCCCUAAAGCCUUCCTT |
| si*CENPM*-2 | GAUCGUGUUUGUGGUUAAUTT | AUUAACCACAAACACGAUCTT |
| sh*Scramble* | TTCTCCGAACGTGTCACGT |  |
| sh*CENPM* | GGAAGGCTTTAGGGCCACC |  |
| *CENPM* | CTGGCGGACTCGATGCTCAAAG | CGATTCACACTGGAGGGCAAAGG |
| *GAPDH* | ACAACAGCCTCAAGATCATCAG | TCTTCTGGGTGGCAGTGATG |
