# Supplementary material for: Identification of CENPM as a key gene driving adrenocortical carcinoma metastasis via physical interaction with immune checkpoint ligand FGL1
Source: Clin Transl Med. 2025 Jan 8;15(1):e70182. doi: 10.1002/ctm2.70182 (PMC11707433; doi:10.1002/ctm2.70182)
Supplement: Supplementary file 3 — Supporting Information [file CTM2-15-e70182-s001.docx]

**Table S3. Antibodies used in this study**

| **Primary Antibodies** | **Catalog Number** | **Applications and Dilutions** | **company** |
| --- | --- | --- | --- |
| IgG | A7016 | IHC, IF 1:90 1:50 | Beyotime, China |
| IgG | AF028 | IF 1:500 1:129 | Beyotime, China |
| IgG | 3900S | Co-IP 4.0 ug for 1.2mg total protein | CST, USA |
| CENPM | DF2314 | IHC, IF 1:50 | Affinity, USA |
| CENPM | PA5-100720 | WB 1:1000 | Invitrogen, USA |
| COL2A1 | Sc-52658 | IF 1:50 | Santa Cruz, USA |
| FGL1 | 16000-1-AP | IF 1:50 WB 1:1000 | Proteintech, China |
| FGL1 | 67391-I-Ig | IF 1:50 | Proteintech, China |
| FGL1 | 16000-1-AP | Co-IP 4.0 ug for 1.2mg total protein | Proteintech, China |
| CILP | bs-13954R | WB: 1:1000 | Bioss, China |
| β-Tubulin | 380628 | WB 1:5000 | ZENBIO, China |
| β-Actin | 380624 | WB 1:5000 | ZENBIO, China |
